# Adjunct Culture of Non-Starter Lactic Acid Bacteria for the Production of Provola Dei Nebrodi PDO Cheese: In Vitro Screening and Pilot-Scale Cheese-Making

**DOI:** 10.3390/microorganisms9010179

**Published:** 2021-01-15

**Authors:** Cinzia Lucia Randazzo, Luigi Liotta, Maria De Angelis, Giuseppe Celano, Nunziatina Russo, Koenraad Van Hoorde, Vincenzo Chiofalo, Alessandra Pino, Cinzia Caggia

**Affiliations:** 1 Department of Agricultural, Food and Environment, University of Catania, 95123 Catania, Italy; cinzia.randazzo@unict.it (C.L.R.); nunziatinarusso83@gmail.com (N.R.); ccaggia@unict.it (C.C.); 2 Department of Veterinary Sciences, University of Messina, 98168 Messina, Italy; luigi.liotta@unime.it; 3 Department of Soil, Plant and Food Science, University of Bari Aldo Moro, 70121 Bari, Italy; maria.deangelis@uniba.it (M.D.A.); giuseppe.celano@uniba.it (G.C.); 4 Department of Foodborne Pathogens, Sciensano, 1050 Brussels, Belgium; Koenraad.VanHoorde@sciensano.be; 5 Department of Chemical, Biological, Pharmaceutical and Environmental Sciences, University of Messina, 98168 Messina, Italy; vincenzo.chiofalo@unime.it

**Keywords:** traditional cheese, autochthonous strains, aminopeptidase activity, flavour formation, volatile organic compounds

## Abstract

The present study aimed at selecting non-starter lactic acid bacteria strains, with desirable technological and enzymatic activities, suitable as adjunct culture for the Provola dei Nebrodi cheese production. One hundred and twenty-one lactic acid bacteria, isolated from traditional Provola dei Nebrodi cheese samples, were genetically identified by Rep-PCR genomic fingerprinting, using the (GTG)_5_-primer, and by matrix-assisted laser desorption/ionisation time-of-flight mass spectrometry (MALDI-TOF MS). Twenty-seven strains, included in the qualified presumption of safety (QPS) list, were tested for technological and proteinase/peptidase activities. Results showed that technological features and flavour formation abilities were strain-dependent. Among the selected strains, *Lacticaseibacillus paracasei* PN 76 and *Limosilactobacillus fermentum* PN 101 were used as adjunct culture in pilot-scale cheese-making trials. Data revealed that adjunct cultures positively affected the flavour development of cheese, starting from 30 days of ripening, contributing to the formation of key flavour compounds. The volatile organic compound profiles of experimental cheeses was significantly different from those generated in the controls, suggesting that the selected adjunct strains were able to accelerate the flavour development, contributing to a unique profile of Provola dei Nebrodi cheese.

## 1. Introduction

The Provola dei Nebrodi (PN) cheese is a historical Sicilian cheese, which has been recognized as a Protected Designation of Origin (PDO) cheese (GUUE L 309—23 September 2020). It is a full-fat raw milk semi-hard pasta filata cheese, produced in the Nebrodi Mountains area under traditional practices, using cow milk or a mix of cow, ewe and goat milk. Lamb or kid rennet paste is used for coagulation, and any starter culture is added during the cheese-making. The PN cheese is generally consumed as a fresh (less than 30 days old), semi-hard (30–120 days old), or hard (more than 120 days old) cheese-type and has a typical pear-like shape with a smooth, shiny, thin, yellow-gold surface and compact straw-coloured core. It is widely recognized that artisanal cheeses, produced under traditional practices using raw milk and without starter additions, boast unique and more intense sensorial characteristics than pasteurized milk cheeses. Their distinctive taste and flavour, greatly appreciated by consumers around the world, is mainly due, along with the environmental conditions and the cheese-making technology applied, to the lactic acid bacteria population (LAB) involved during the cheese production and ripening [1,2,3]. In particular, starter LAB (SLAB) are implicated in the lactose fermentation, producing high concentrations of lactic acid and enhancing the curd acidification, whereas non-starter LAB (NSLAB) are present during the cheese ripening, influencing the biochemistry of curd maturation and contributing to the development of the sensorial characteristics of the final product [4,5]. The NSLAB population plays a key role in cheese flavour compound development through three metabolic pathways: the metabolism of lactate and citrate, the release of free fatty acids and their subsequent metabolism, and the protein breakdown and amino acids catabolism [6,7,8]. It is well known that several NSLAB strains possess a comprehensive proteinase/peptidase system which include, besides the cell envelope-associated proteinase PrtP, several intracellular oligoendopeptidases (PepO, PapF, PepP), general aminopeptidases (PepN, PepC, PepG), glutamylaminopeptidase PepA, leucyl-aminopeptidase PepL, prolyl-dipeptidyl aminopeptidase PepX, tripeptidase (PepT), dipeptidase (PepQ, PepV) and several further proteinases/peptidases [9,10]. These proteinase/peptidase systems strongly influence the sensorial characteristics of both semi-hard and hard cheeses [11]. In particular, during cheese ripening, PepN positively influences both the saltiness and maturity indexes, and it is generally responsible for a reduction in sweetness and bitterness aromatic compounds. In addition, PepL is responsible for cheesy, chocolate, sweaty and malt aromas, whereas proline-aminopeptidase (PepI) is considered a good indicator of pronounced sourness [11]. In addition, NSLAB harbour a complex system of amino acid catabolizing enzymes [12]. Therefore, to ensure the enhancement of sensory properties of cheese, NSLAB strains, which can be used as adjunct cultures, should be carefully selected based on both technological parameters and enzymatic activities.

The present study aimed to select NSLAB strains to be used as adjunct cultures during the PN cheese production. To this end, NSLAB strains were isolated, genotypically characterized, and studied for their technological properties. Subsequently, selected strains were used in the PN cheese-making in order to validate, at pilot scale, their efficacy in the proteolytic metabolism during ripening.

## 2. Materials and Methods

### 2.1. Cheese Samples and NSLAB Isolation

The artisanal Provola dei Nebrodi PDO cheese (PN) samples, analysed in the present study, were produced under traditional practices in three different farms (I, II, and III) located in the surrounding area of the Nebrodi Mountains (Sicily, Italy). For each farm, semi-hard cheese samples (60 days old), obtained from three independent batches, were collected and transferred to the Laboratory of Microbiology (Department of Agricultural, Food, and Environment, University of Catania, Italy), under refrigerated conditions (4 ± 2 °C). Overall, 9 PN cheese samples (both core and surface sections) were subjected to NSLAB isolation according to Pino et al. [13], using de Man Rogosa and Sharpe (MRS) (Oxoid, Milan, Italy) agar medium, adjusted to pH 5.4, incubated at 32 °C for 72 h (5 days) under microaerophilic conditions. Cycloheximide 100 mg/mL (Sigma, Milan, Italy), was added to inhibit yeast growth. From each MRS agar plate, about 20% of the colonies were randomly selected, checked for morphology, catalase activity, and Gram reaction, then purified, by streaking three times on MRS agar plates. Pure cultures were stored in liquid culture using 20% glycerol at −80 °C.

### 2.2. NSLAB Identification

Total genomic DNA was extracted from NSLAB isolates by mechanical cell lysis following the protocol previously described [14]. DNA concentration was evaluated by measuring optical density using a Fluorometer Qubit (Invitrogen, Carlsbad, CA, USA). NSLAB identification was performed by Rep-PCR genomic fingerprinting, using a (GTG)_5_-primer, and matrix-assisted laser desorption/ionisation time-of-flight mass spectrometry (MALDI-TOF MS) (Bruker Daltonics, Germany) according to Nguyen et al., and Pino et al. [15,16].

### 2.3. Safety Assessment

#### 2.3.1. DNAse, Gelatinase, Haemolytic Activities and Mucin Degradation Ability

NSLAB strains were evaluated for DNAse and gelatinase activities according to Lavilla-Lerma et al. [16]. Mucin degradation ability was tested following the method reported by Muñoz-Atienza et al. [17]. Haemolytic activity was evaluated on blood agar plates containing sheep blood (Biolife, Milan, Italy) according to Pino et al. [18]. The haemolytic positive strains *Streptococcus pyogenes* ATCC 19,615 and *Streptococcus pneumoniae* ATCC 6303, cultured on Brain-Heart Infusion (BHI, Merck, Italy) at 37 °C under 5% of CO_2_, were used as positive controls.

#### 2.3.2. Biogenic Amine Production

The ability of the strains to produce biogenic amines by amino acid decarboxylation was tested according to Bover-Cid and Holzapfel [19] using tyrosine (freebase), histidine monohydrochloride, ornithine monohydrochloride or lysine monohydrochloride as precursor amino acids. Plates without amino acids were used as controls. All amino acids were purchased from Sigma.

#### 2.3.3. Antibiotic Susceptibility and Minimum Inhibitory Concentration Determination

NSLAB isolates were evaluated for antibiotic susceptibility according to breakpoints proposed by the European Food Safety Authority (EFSA) [20]. The minimum inhibitory concentration (MIC) was determined by micro-dilution method according to Russo et al. [21]. Strains showing MICs lower or higher than the EFSA’s breakpoints were considered as sensitive or resistant, respectively [20].

#### 2.3.4. Genes for Virulence, Biogenic Amines, and Antibiotic Resistance

A PCR-based approach was applied to investigate the presence of gene encoding virulence factors, biogenic amines production, and antibiotic resistance as reported by Pino et al. [16]. The amplicons were separated by electrophoresis on 0.8 to 2.0% (*w*/*v*) agarose gels in 0.5× TAE buffer, and gels were stained in 0.5× TAE buffer using the GelRed^®^ Nucleic Acid Gel Stain (Biotium, Fremont, CA, USA).

### 2.4. Technological Features

#### 2.4.1. Salt Tolerance

The lactobacilli strains, fulfilling the aforementioned safety properties, were tested for the ability to grow in saline solutions containing 2, 6, and 10% (*w*/*v*) of NaCl as reported by Ferrari et al. [22].

#### 2.4.2. Proteolytic and Lipolytic Activities, Exopolysaccharides and Diacetyl Production

Proteolytic and lipolytic activities were evaluated according to Meng et al. [1] using Plate Count Agar supplemented with 1% (*w*/*v*) skim milk powder (Oxoid, Milan, Italy) and Tributyrin Agar (Merck, France) media, respectively. Exopolysaccharides (EPS) production was tested on both MRS and mMRS, in which glucose was replaced by 10% of sucrose, following the method described by Meng and co-workers [1]. The NSLAB strains’ ability to produce diacetyl was evaluated according to Ribeiro et al. [23]. Diacetyl production was indicated by the formation of a red ring at the top of the tubes. Based on the presence and intensity of the red colour, the strains were scored as no (−), moderate (+), high (++), or strong (+++) diacetyl producers.

#### 2.4.3. Acidifying Activity

To test acidifying activity, overnight cell cultures were grown in MRS broth at 37 °C, standardized to 9 log cfu/mL, and inoculated (2% *v*/*v*) into skim milk (Oxoid, Milan, Italy). The pH changes (ΔpH) were determined after 6–8 h of incubation at 37 °C using a pH meter (H19017, Microprocessor, Hanna Instruments, Ronchi di Villafranca Padovana, Italy).

#### 2.4.4. Peptidase Activities

Aminopeptidase activities were evaluated on cell free extract (CFE) obtained from stationary phase cell cultures by lysozyme cell-lysis [24]. The p-nitroanilide (pNA) substrates Ala-pNA, Leu-pNA, Lys-pNA, Glu-pNA, and Pro-pNA were used according to González et al. [24]. One unit of aminopeptidase activity was considered as the enzyme amount able to determine an increase in absorbance of 0.001 units. Aminopeptidase activity was expressed as the number of activity units per milligram of protein per minute. The analyses were performed in triplicate and results are reported as mean and standard deviation.

### 2.5. Experimental Pilot Scale Cheese-Making

Two pilot-scale cheese-making trials, experimental (ECh) and control (CCh), were carried out in triplicate (three repetitions of each trial were conducted at different days) in a local dairy factory following the traditional cheese-making procedure and using 150 L of cow’s milk. The experimental Provola dei Nebrodi cheeses (ECh) were obtained by adding the *Lacticaseibacillus paracasei* PN 76 and the *Limosilactobacillus fermentum* PN 101 strains, which were selected based on safety and technological properties as previously described. The aforementioned stains, used as an adjunct culture at a cell density of 10^8^ cfu/mL, were inoculated in a 1:1 ratio before milk coagulation. The control Provola dei Nebrodi cheeses (CCh) were obtained without any adjunct culture. Both experimental (ECh) and control (CCh) cheeses were collected at 0, 30 and 60 days of ripening and were subjected to microbiological and physico-chemical analyses. For each trial, samples were collected and analysed in triplicate.

### 2.6. Microbiological Analysis

A mixture of both core and surface sections (25 g) of the ECh or CCh samples at 0, 30, and 60 days of ripening were subjected to total mesophilic bacteria, lactic acid bacteria, Enterobacteria, lactococci, yeasts, *Listeria* spp., *Escherichia coli,* faecal coliforms and staphylococci counts following the method and using the culture media reported by Pino et al. [14]. In addition, Kanamycin Aesculin Azide agar (KAA), incubated under anaerobic conditions at 37 °C for 24–48 h, was used for enterococci count. All media were purchased from Oxoid (Basingstoke, UK).

### 2.7. Physico-Chemical Analysis

A mix of both core and surface cheese sections (100 g) of CCh or ECh samples at 0, 30 and 60 days of ripening were analysed for moisture, protein, fat, and salt content, using near-infrared spectroscopy in transmittance (FoodScanTM Dairy Analyser; FOSS, Hilleroed, Denmark). The ash content was determined following the Association of Official Analytical Chemists (AOAC) method (942.05 1942) [25]. In addition, the pH value of each sample was determined by pHmeter (H19017, Microprocessor, Hanna Instruments). Each analysis was conducted in triplicate and results are expressed in g/100 g of edible part. The analyses were performed at the Laboratory of the Animal Production Unit, Department of Veterinary Sciences, University of Messina, Italy.

### 2.8. Analysis of Volatile Organic Compounds (VOCs)

Four grams of grated cheese, supplemented with 10 µL of internal standard solution 4-methyl-2-pentanol (33 ppm) were placed into 20 mL glass vials and sealed with polytetrafluoroethylene (PTFE)-coated silicone rubber septa (20 mm diameter) (Supelco, Bellefonte, PA, USA). In order to obtain the best extraction efficiency, the micro-extraction procedure was performed as described by Delgado et al. [26], with slight modifications. At the end of sample equilibration (10 min at 50 °C), a conditioned 50/30 μm DVB/CAR/PDMS fibre (Supelco, Bellefonte, PA, USA) was exposed to headspace at the same temperature for 60 min to obtain VOC adsorption. To keep the temperature constant during analysis, the vials were maintained on a heater plate (CTC Analytics, Zwingen, Switzerland) and the extraction was carried out by a CombiPAL system injector autosampler (CTC Analytics). The extracted compounds were desorbed in splitless mode for 3 min at 220 °C and analysed by Clarus 680 (Perkien Elmer) gas-chromatography equipped with a capillary column Rtx-WAX column (30 m × 0.25 mm i.d., 0.25 μm film thickness) (Restek, Bellfonte, PA, USA). The column temperature was set initially at 35 °C for 8 min, then increased to 60 °C at 4 °C min^−1^, to 160 °C at 6 °C min^−1^ and finally to 200 °C at 20 °C min^−1^ and held for 15 min [27]. Helium was used as the carrier gas at a flow rate of 1 mL min^−1^. The analyses lasted for 50 min. The single quadrupole mass spectrometer Clarus SQ 8C (Perkien Elmer) was coupled to the gas chromatography system. The source and transfer line temperatures were kept at 250 and 230 °C, respectively. Electron ionisation masses were recorded at 70 eV, and the mass-to-charge ratio interval was 34 to 350 *m/z*. The GC-MS generated a chromatogram with peaks representing individual compounds. Each chromatogram was analysed for peak identification using the National Institute of Standard and Technology 2008 (NIST) library. A peak area threshold >1,000,000 and 85% or greater probability of match was used for VOC identification followed by manual visual inspection of the fragment patterns when required. The concentrations of VOCs, expressed as mg/kg, were calculated by using the internal standard.

### 2.9. Statistical Analysis

Acidifying activity, microbiological data, and VOC concentrations were subjected to ANOVA (one-way analysis of variance) followed by Tukey’s post-hoc test. Statistical analysis was performed using XLSTAT PRO 5.7 (Addinsoft, New York, NY, USA) setting the reference level of significance to 0.05 in all assays. In addition, VOC data were subjected to permutation analysis using PermutMatrixEN software (Montpellier, France).

## 3. Results

### 3.1. NSLAB Identification

**Figure 1:**
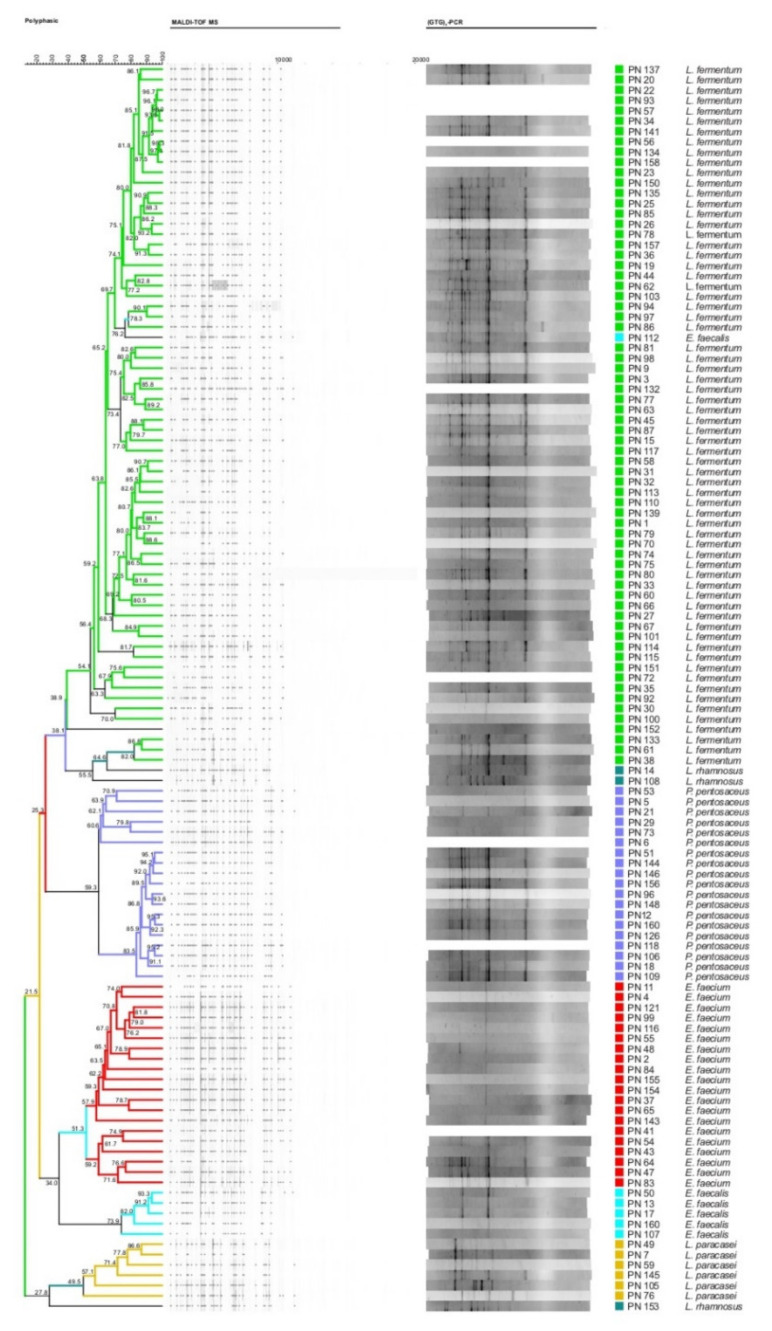
UPGMA dendrogram of the Rep-PCR and matrix-assisted laser desorption/ionisation time-of-flight mass spectrometry (MALDI-TOF MS) analyses of presumptive non-starter lactic acid bacteria (NSLAB) isolated from semi-hard Provola dei Nebrodi PDO (PN) cheese samples. Node values indicate the average percentage of similarity based on (GTG)_5_-PCR and MALDI-TOF MS profiles. The tree was made with BioNumerics version 5.1.

Overall, 160 isolates were obtained and 121 of them were found to be Gram-positive, catalase-negative, rod or coccal in shape, non-motile, and unable to form spores. As reported in Figure 1, REP-PCR analysis coupled to MALDI-TOF MS allowed the classification of them into six species. Overall, the isolates were ascribed to *Limosilactobacillus fermentum* (55%), *Enterococcus faecium* (17%), *Pediococcus pentosaceus* (16%), *Lacticaseibacillus paracasei* (5%), *Enterococcus faecalis* (5%), and *Lacticaseibacillus rhamnosus* (2%) species.

### 3.2. Safety Properties

Ninety-five (95) isolates, with the exception of those belonging to *E. faecium* and *E. faecalis* species, not included in the qualified presumption of safety (QPS) list, were screened for safety requirements. None of the tested strains showed the ability to produce DNAse and gelatinase, to degrade the mucin, and to exert haemolytic activity. Three strains (PN 6, PN 51, and PN 18), ascribed to *P. pentosaceus* were able to produce biogenic amines (BA) by the decarboxylation of tyrosine. NSLABs were susceptible to tested antibiotics with the exception of *P. pentosaceus* PN 21, PN 29, PN 73, PN 96, PN 118, and PN 126 strains which exhibited, for both gentamicin and erythromycin, MIC values higher than the EFSA breakpoints (Table 1). The PCR-based approach revealed the presence of genetic determinants conferring resistance to gentamicin and erythromycin only for the isolates exhibiting MIC values higher than the EFSA breakpoints. In addition, none of the tested isolates carried genes encoding for virulence factors and amino acid decarboxylation.

### 3.3. Technological Features

#### 3.3.1. Salt Tolerance

Sodium chloride tolerance exhibited by the tested NSLAB isolates is reported in Table 2. Overall, all stains were able to tolerate 2% of NaCl. The ability to grow in the presence of 6% of NaCl was exhibited by 70 strains (56 *L. fermentum*, 5 *P. pentosaceus*, 6 *L. paracasei*, and 3 *L. rhamnosus*), whereas 27 strains (20 *L. fermentum*, 5 *L. paracasei*, and 2 *L. rhamnosus*) tolerated a NaCl concentration of 10%.

#### 3.3.2. Proteolytic and Lipolytic Activities, Exopolysaccharides and Diacetyl Production Abilities

Table 2 shows the extracellular proteolytic and lipolytic activities, exopolysaccharides (EPS) and diacetyl production abilities exhibited by the 86 tested NSLABs. Regarding proteolytic activity, 36 isolates (42%) showed transparent halo-forming colonies when grown on Plate Count Agar supplemented with 1% (*w*/*v*) skim milk powder. In particular, all the isolates ascribed to *L. paracasei* and *L. rhamnosus* species exhibited proteolytic activity, as well as 22 *L. fermentum* and 5 *P. pentosaceus* strains (Table 2). Lipolytic activity, assayed on tributyrin agar (TBA), was shown by 14 strains (16%). Among these, nine belonged to *L. fermentum*, three were ascribed to *P. pentosaceus*, and two were attributed to *L. rhamnosus* species. None of the *L. paracasei* isolates showed positive lipolytic capacity on the TBA medium. As reported in Table 2, 26 *L. fermentum*, 5 *L. paracasei* and 3 *L. rhamnosus* isolates were positive for EPS phenotypes, whereas none of the isolates ascribed to *P. pentosaceus* showed colonies with a slimy appearance. The ability to produce diacetyl from citrate was exhibited by 29 strains (34%) (Table 2). Among these, 22 *L. fermentum*, 5 *L. paracasei* and 2 *L. rhamnosus* isolates exhibited high or strong diacetyl-producing capabilities.

#### 3.3.3. Acidifying Activity

**Figure 2:**
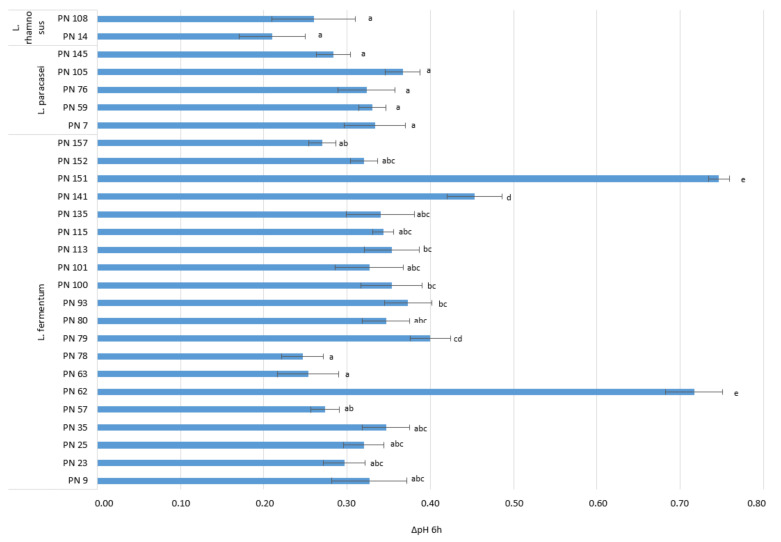
Acidification activity of the selected NSLAB strains isolated from artisanal semi-hard Provola dei Nebrodi PDO cheese samples. Results are reported as mean and standard deviation of three replicates. For each species, bars with different lowercase letters (**a**–**e**) are significantly different at *p* < 0.05.

The acidification activity of the isolates, selected based on the aforementioned technological properties, is reported in Figure 2. Overall, all the isolates showed slow acidification activity determining, after 6–8 h of incubation, a drop in pH values ranging from 0.24 to 0.75.

#### 3.3.4. Peptidase Activities

Based on the aforementioned technological properties, 27 strains were tested for aminopeptidase activities, and results are reported in Table 3. Overall, the highest degree of aminopeptidase activity was recorded against the Lys-pNA substrate (range 120–0.42 activity units per milligram of protein per minute), whereas the lowest values were observed using the Ala-pNA substrate (range 2.29–0.16 activity units per milligram of protein per minute). All strains showed aminopeptidase activity towards the tested substrates with the exception of *L. fermentum* PN 135 and *L. paracasei* PN 105, which exhibited undetectable aminopeptidase activity towards both the Lys-pNA and the Glu-pNA substrates. *L. fermentum* PN 101 exhibited the highest aminopeptidase activity towards both Lys-pNA and Ala-pNA substrates, whereas the *L. paracasei* PN 76 stain appeared promising, showing the highest aminopeptidase activity toward both Leu-pNA and Pro-pNA substrates.

### 3.4. Microbiological Analysis of Control and Experimental Cheeses

Microbiological data of ECh and CCh cheese samples at 0, 30 and 60 days of ripening, expressed as average values and standard deviations, are reported in Table 4. Regarding ECh samples, mesophilic aerobic bacteria, lactic acid bacteria, and lactococci showed a decreasing trend throughout the ripening time with a reduction of about 1 log unit at 60 days of ripening. Enterobacteria, staphilococci, yeasts and enterococci exhibited a quite constant value up to 60 days of ripening. With regards to CCh samples, mesophilic aerobic bacteria, enterobacteria, and staphilococci showed a significant reduction at 60 days of ripening, whereas the number of lactobacilli, lactococci, yeasts, and enterococci revealed constant cell densities throughout the whole ripening time. Evaluating differences between ECh and CCh samples, statistical data revealed that the addition of adjunct cultures significantly influenced the level of lactobacilli and lactococci count, which showed, from the beginning of the ripening, higher values on ECh samples (Table 4). Moreover, the level of enterobacteria was significantly reduced by the addition of the adjunct cultures at both 0 and 30 days of ripening. *Listeria* spp., *Escherichia coli* and faecal coliforms were never detected in the analysed samples (Table 4).

### 3.5. Physico-Chemical Analysis

The physico-chemical composition of experimental (ECh) and control (CCh) cheese samples at 0, 30 and 60 days of ripening are reported in Table 5. Overall, at the same time of ripening, no significant differences were recorded among the analysed samples.

### 3.6. Volatile Organic Compounds (VOCs) Detection

**Figure 3:**
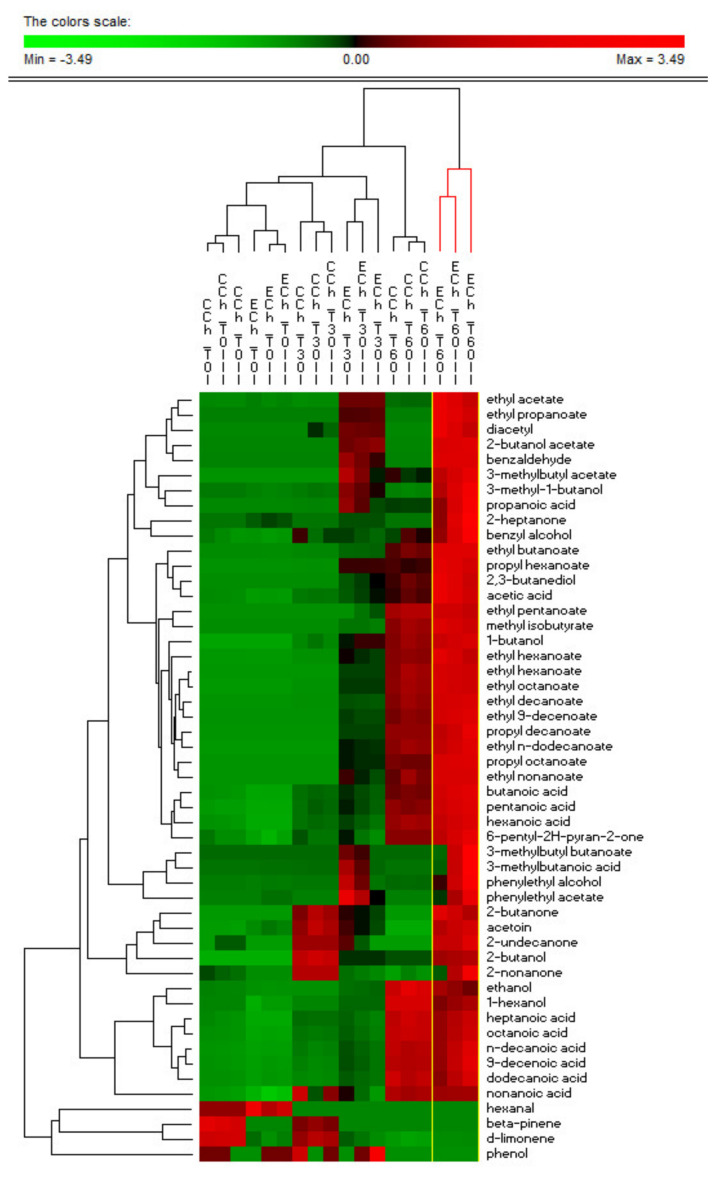
Grouping of samples based on the volatile organic compounds (VOCs) identified in experimental (ECh) and control (CCh) cheeses at 0, 30 and 60 days of ripening. The different batch of cheeses are indicated as I, II and III.

The VOCs detected in ECh and CCh cheese samples at 0, 30, and 60 days of ripening are reported in Table 6. In addition, similarities in the observed metabolomics profiles between ECh and CCh samples, estimated using the PermutMatrixEN software, are displayed in Figure 3. Overall, the analysis allowed the identification of 51 compounds as alcohols, aldehydes, esters, ketones, organic acids, and terpenes. Organic acids represented the main VOCs detected, whereas aldehydes were the less abundant. Total VOCs exhibited a growing trend during the ripening time in all the analysed samples, reaching the highest value in ECh samples at 60 days of ripening (219.34 mg/kg). Focusing on each chemical class, statistically significant differences were recorded among samples. In particular, among organic acids, hexanoic acid, butanoic acid, acetic acid, propanoic acid, and pentanoic acid were detected at higher concentrations in ECh samples at 60 days of ripening. Similarly, among alcohols, phenylethyl alcohol, benzyl alcohol, and 2,3-butanediol were mainly detected in ECh samples at 60 days of ripening, whereas 3-methyl-1-butanol, 2-butanol, and 1-butanol reached the highest concentration in ECh samples, at both 30 and 60 days of ripening. Ethyl hexanoate, ethyl octanoate, ethyl butanoate, ethyl decanoate, and ethyl acetate were significantly different among ECh and CCh samples at both 30 and 60 days of ripening, whereas ethyl propanoate, 2-butanol acetate, phenylethyl acetate, and 3-methylbutyl butanoate were detected only in ECh samples at 30 and 60 days of ripening. Among ketones, 2-butanone, diacetyl, acetoin, and 2-undecanone were significantly different among ECh and CCh samples at 30 days of ripening. In addition, the aforementioned ketones were not detected in ECh samples at 60 days of ripening. Among terpenes, beta-pinene was revealed only in CCh samples at 0 and 30 days of ripening.

## 4. Discussion

It is already well established that selected NSLAB are mainly used as adjunct culture in cheese production for their ability to contribute to flavour development, affecting the sensorial characteristics of the final product [8,28,29,30]. The main metabolic activities, which contribute to cheese flavour development, are glycolysis, lipolysis, and proteolysis. In particular, catabolic products of proteolytic reactions represent the major metabolic pathway, which contribute to flavour development [29,30,31]. According to that, peptidase activity of lactobacilli has been widely investigated [29,30,32].

In the present study, 121 strains were considered LAB and only those with safety properties (86) were tested for technological features, showing a strain-dependent profile in accordance to previously reported data [33,34,35,36]. In addition, 27 selected strains were subjected to aminopeptidase tests such as the Pro-p-NA, which is related to the ability to release high quantities of proline from caseins during cheese ripening [37,38]; Pep L, Pep N, and Pep M, which, thanks to the release of amino acids in the cheese environment, serve as precursors of several flavour compounds [38,39]. The screening of the isolates allowed us to select the *Lacticaseibacillus paracasei* PN 76 and the *Limosilactobacillus fermentum* PN 101 strains as promising adjunct cultures in a pilot-scale cheese manufacture. In fact, as well as satisfying the safety requirements, both strains showed interesting technological properties (proteolytic activity, abilities to produce both exopolysaccharides and diacetyl). In addition, based on the peptidase activity, the *L. fermentum* PN 101 exhibited the highest aminopeptidase activity towards both Lys-pNA and Ala-pNA substrates, whereas the *L. paracasei* PN 76 strain showed the highest aminopeptidase activity toward both Leu-pNA and Pro-pNA substrates. As expected, physico-chemical compositions of both experimental and control cheeses were quite similar through ripening, while significant differences were revealed comparing the GC–MS profile. In fact, the experimental cheeses at 30 and 60 days of ripening revealed key flavour compounds which were not detected in the controls. Lipolysis of the triglycerides by indigenous milk lipase, microbial esterase, and enzymes from rennet mainly resulted in the production of medium-chain (hexanoic and octanoic acid) and long-chain free fatty acids (FFAs) [40]. In particular, the experimental cheeses exhibited high concentrations of hexanoic, butanoic, and acetic acids. Although lipoprotein lipase activity is of the utmost importance in cheese varieties made from raw milk, the presence of NSLAB enzymes over an extended period can lead to the liberation of significant FFA levels in cheese [40,41,42]. According to Stefanovic et al. [43], *L. paracasei* strains could contribute to the development and diversification of metabolites of lipid origin, such as long chain aldehydes, acids, ketones, and lactones. Among the aforementioned compounds, hexanoic acid, which generates popcorn, sharp, goaty, burnt, and waxy aromas [44,45], has previously been revealed in Pecorino Siciliano PDO cheese, Pecorino Crotonese, Grana Padano, Roncal, Caciocavallo Palermitano and Gouda-type cheeses [11,33,34,46,47,48,49,50] older than 60 ripening days. Similarly to other cheese types, such as Cheddar, Gruyère, Roncal, and Emmental [6,11,46,47,48,49,50], organic acids, important aromatic compounds, as well as precursors of methyl ketones, alcohols, lactones and esters, were the main detected VOCs. In addition, propanoic acid, with a typical vinegary odour, and pentanoic acid, responsible for rain, wood, vegetable, spicy, nutty, and grain aromas, were mainly identified in 60-day ripened experimental cheeses. Some differentiation in volatile profiles occurred based on differences in VOCs arising from FAA metabolism. It is interesting to note that the 3-methylbutanoic acid, a carboxylic acid characteristic of goat and sheep cheeses, appeared only in experimental cheeses. Notably, this organic acid, related to an extensive breakdown of proteins and probably derived from leucine catabolism, confers cheesy, sweaty, and very-ripened cheese aromas [6,11]. Similarly to other cheese-types, such as Parmigiano [50], Canestrato Pugliese cheese [51,52], Grana-type cheeses [50], Gouda-type cheese varieties [48], and other cheese varieties [53], esters were the second major class of detected VOCs. Esters confer sweet, fruity, and floral notes, and contribute to the aroma of cheese by reducing the sharpness and the bitterness perceptions provided by fatty acids and amines, respectively [11,54]. Regarding to the alcohol profile of experimental cheeses, both primary and secondary alcohols were detected similarly to other cheese types, such as Canestrato Pugliese [51,52], Cheddar [45], Manchego [55] and Roncal [56]. Particularly high level of 3-methyl 1-butanol, phenylethyl alcohol, and benzyl alcohol were detected in experimental cheese at 60 days of ripening. The 3-methyl 1-butanol, which confers an appreciated aroma of fresh cheese, often associated to a fruity perception [33,34], is considered an important contributor to overall flavour. This secondary alcohol is related to the degradation of branched-chain amino acids during cheese ripening, and its presence indicates the reduction of the aldehyde produced from leucine [11]. Regarding the ketones, it is interesting to note that this class of VOCs was revealed only in experimental cheeses. The FFAs’ contribution to cheese flavour is not only direct but also indirect, because they are precursors of methyl ketones and secondary alcohols [57]. Ketones derive from the β-oxidation of fatty acids and are normally present in dairy products. Among these, 2-butanone, responsible for butterscotch aromas and diacetyl were detected in experimental cheeses at high levels.

## 5. Conclusions

In conclusion, the screening of the autochthonous NSLABs, isolated from the Provola dei Nebrodi PDO cheese, based on technological and aminopeptidase properties, allowed the selection of the *Lacticaseibacillus paracasei* PN 76 and the *Limosilactobacillus fermentum* PN 101 strains as promising adjunct cultures. Their use in pilot-scale cheese-making trials highlighted a consistent influence on flavour development, starting from 30 days of ripening, contributing to the formation of key flavour compounds.

## Figures and Tables

**Table 1:** Minimum inhibitory concentration (MIC) values showed by NSLAB strains obtained from 60-day ripened Provola dei Nebrodi PDO cheese samples.

Antimicrobials	Species (No. of Tested Isolates)	Number of Strains which Showed the Indicated MIC Value (mg/L) ^a^															Breakpoints (mg/L) ^b^
		0.06	0.12	0.25	0.5	1	2	4	8	16	32	64	128	256	512	1024	
Ampicillin	*L. fermentum* (67)		7	13	23	24											2
	*P. pentosaceus* (16)				13	3											4
	*L. paracasei* (6)			1	5												4
	*L. rhamnosus* (3)			3													4
Gentamicin	*L. fermentum* (67)				15	11	41										16
	*P. pentosaceus* (16)						2	3	5			6					16
	*L. paracasei* (6)						2	4									32
	*L. rhamnosus* (3)								3								16
Kanamycin	*L. fermentum* (67)						3	21		43							32
	*P. pentosaceus* (16)							6	10								64
	*L. paracasei* (6)									6							64
	*L. rhamnosus* (3)							3									64
Streptomycin	*L. fermentum* (67)							13	21	18	15						64
	*P. pentosaceus* (16)								16								64
	*L. paracasei* (6)								1	5							64
	*L. rhamnosus* (3)						1	2									32
Erythromycin	*L. fermentum* (67)	15	21	28													1
	*P. pentosaceus* (16)		4	6				2		4							1
	*L. paracasei* (6)		4	2													1
	*L. rhamnosus* (3)		2	1													1
Clindamycin	*L. fermentum* (67)			14	53												1
	*P. pentosaceus* (16)		12		4												1
	*L. paracasei* (6)		4														1
	*L. rhamnosus* (3)			3													1
Tetracycline	*L. fermentum* (67)				51	16											8
	*P. pentosaceus* (16)						16										8
	*L. paracasei* (6)			6													4
	*L. rhamnosus* (3)				3												8
Chloramphenicol	*L. fermentum* (67)					51	16										4
	*P. pentosaceus* (16)			11	5												4
	*L. paracasei* (6)				6												4
	*L. rhamnosus* (3)			2	1												4

^a^ MICs determined by micro-dilution method. ^b^ Breakpoints proposed by the European Food Safety Authority (EFSA). The following antibiotic dilution ranges were tested: 0.12–64 mg/L for ampicillin; 1–512 mg/L for gentamicin; 2–1024 for both kanamycin and streptomycin; 0.06–1 mg/L for erythromycin and clindamycin; 0.25–128 mg/L for tetracycline; 0.25–64 mg/L for chloramphenicol. Isolates with MICs higher than the EFSA breakpoints, indicated in bold, were considered as resistant.

**Table 2:** Salt tolerance, proteolytic and lipolytic activities, exopolysaccharides (EPS) and diacetyl production exhibited by the NSLAB strains isolated from artisanal semi-hard Provola dei Nebrodi PDO (PN) cheese samples.

Species	Strains	Salt Tolerance *			Proteolytic Activity *	Lipolytic Activity *	EPS Production *	Diacetyl Production **
		2%	6%	10%				
*Limosilactobacillus fermentum* (*n* = 67)	PN 1; PN 3; PN 22; PN 26; PN 30; PN 31; PN 33; PN 34; PN 36; PN 44; PN 45; PN 56; PN 58; PN 61; PN 66; PN 70; PN 72; PN 75; PN 77; PN 85; PN 86; PN 87; PN 92; PN 97; PN 98; PN 103; PN 134; PN 137; PN 139; PN 150.	+	+	−	−	−	−	−
	PN 9; PN 23; PN 25; PN 35; PN 57; PN 62; PN 63; PN 78; PN 79; PN 80; PN 93: PN 100; PN 101; PN 113; PN 115; PN 135; PN 141; PN 151; PN 152; PN 157.	+	+	+	+	−	+	++
	PN 15; PN 20; PN 27; PN 32; PN 67; PN 94; PN 117; PN 132; PN 133.	+	−	−	−	+	−	−
	PN 19; PN 38; PN 60; PN 74; PN 110; PN 114.	+	+	−	−	−	+	−
	PN 81; PN 158.	+	−	−	+	−	−	+
*Pediococcus penstosaceus* (*n* = 10)	PN 5; PN 53; PN 146.	+	−	−	+	+	−	−
	PN 12; PN 109.	+	−	−	+	−	−	−
	PN 16; PN 106; PN 144; PN 148; PN 156.	+	+	−	−	−	−	−
*Lacticaseibacillus paracasei* (*n* = 6)	PN 7; PN 59; PN 76; PN 105; PN 145.	+	+	+	+	−	+	+++
	PN 49.	+	+	−	+	−	−	−
*Lacticaseibacillus rhamnosus* (*n* = 3)	PN 14; PN 108.	+	+	+	+	+	+	++
	PN 153.	+	+	−	+	−	+	−

* (+) positive reaction; (−) negative reaction. ** The strains were scored as no (−), moderate (+), high (++), or strong (+++) diacetyl producer.

**Table 3:** Peptidase activities of the NSLAB strains.

Species	Strain	Pep N	Pep A	Pep L	Pep M	Pep I
*L. fermentum*	PN 9	76.77 ^c^ ± 0.54	0.16 ^hm^ ± 0.04	7.73 ^n^ ± 0.14	19.23 ^j^ ± 0.17	35.63 ^d^ ± 0.36
	PN 23	42.08 ^d^ ± 0.17	0.89 ^efg^ ± 0.07	0.25 ^q^ ± 0.05	24.21 ^fg^ ± 0.13	22.40 ^i^ ± 0.20
	PN 25	0.5 ^l^ ± 0.12	2.29 ^a^ ± 0.09	26.44 ^d^ ± 0.33	27.74 ^d^ ± 0.22	24.77 ^g^ ± 0.18
	PN 35	0.50 ^l^ ± 0.06	0.70 ^eghim^ ± 0.06	43.02 ^a^ ± 0.14	23.57 ^fg^ ± 0.32	20.18 ^k^ ± 0.13
	PN 57	10.63 ^j^ ± 0.48	0.75 ^eghm^ ± 0.08	25.69 ^d^ ± 0.31	26.37 ^e^ ± 0.26	22.54 ^i^ ± 0.16
	PN 62	17.41 ^i^ ± 0.47	1.85 ^b^ ± 0.09	14.11 ^j^ ± 0.13	18.93 ^j^ ± 0.16	7.60 ^q^ ± 0.21
	PN 63	19.45 ^h^ ± 0.46	1.1 ^cf^ ± 0.10	19.27 ^f^ ± 0.36	5.58 ^pq^ ± 0.15	13.28 ^n^ ± 0.14
	PN 78	0.4 7 ^l^ ± 0.12	1.28 ^c^ ± 0.14	17.57 ^h^ ± 0.35	18.37 ^j^ ± 0.15	28.23 ^f^ ± 0.09
	PN 79	18.54 ^hi^ ± 0.38	0.70 ^eghim^ ± 0.07	20.55 ^eg^ ± 0.37	7.71 ^n^ ± 0.16	32.83 ^e^ ± 0.13
	PN 80	6.52 ^k^ ± 0.29	1.85 ^b^ ± 0.10	5.95 ^a^ ± 0.07	30.63 ^c^ ± 0.44	16.87 ^m^ ± 0.26
	PN 93	30.67 ^e^ ± 0.45	1.43 ^cd^ ± 0.10	10.68 ^lm^ ± 0.13	4.57 ^p^ ± 0.36	18.25 ^l^ ± 0.22
	PN 100	0.49 ^l^ ± 0.07	0.79 ^eghm^ ± 0.12	17.37 ^h^ ± 0.35	21.42 ^h^ ± 0.27	10.29 ^a^ ± 0.14
	PN 101	120.75 ^a^ ± 0.32	0.37 ^im^ ± 0.05	30.64 ^b^ ± 0.37	41.83 ^a^ ± 0.17	39.63 ^b^ ± 0.23
	PN 113	0.48 ^l^ ± 0.07	0.71 ^eglm^ ± 0.10	29.26 ^c^ ± 0.05	7.12 ^nq^ ± 0.12	23.74 ^h^ ± 0.18
	PN 115	0.45 ^l^ ± 0.06	0.38 ^hm^ ± 0.06	21.27 ^e^ ± 0.16	16.72 ^k^ ± 0.16	24.67 ^gh^ ± 0.27
	PN 135	ND	ND	10.16 ^m^ ± 0.11	24.51 ^f^ ± 0.23	8.97 ^p^ ± 0.18
	PN 141	0.46 ^l^ ± 0.07	0.67 ^eghm^ ± 0.09	13.07 ^k^ ± 0.16	5.29 ^pq^ ± 0.23	37.63 ^c^ ± 0.34
	PN 151	0.44 ^l^ ± 0.06	0.66 ^ghm^ ± 0.10	13.22 ^j^ ± 0.16	16.65 ^k^ ± 0.14	20.37 ^jk^ ± 0.27
	PN 152	0.46 ^l^ ± 0.16	0.64 ^ghm^ ± 0.06	19.57 ^fg^ ± 0.13	15.07 ^l^ ± 0.16	9.21 ^p^ ± 0.16
	PN 157	0.42 ^l^ ± 0.04	1.68 ^bd^ ± 0.10	0.85 ^pq^ ± 0.09	14.74 ^l^ ± 0.17	19.69 ^k^ ± 0.26
*L. paracasei*	PN 7	0.45 ^l^ ± 0.06	1.19 ^ce^ ± 0.06	7.58 ^n^ ± 0.34	6.14 ^oq^ ± 0.14	21.30 ^j^ ± 0.25
	PN 59	26.54 ^g^ ± 0.45	0.88 ^efg^ ± 0.08	11.57 ^l^ ± 0.28	9.42 ^m^ ± 0.20	19.51 ^k^ ± 0.20
	PN 76	96.65 ^b^ ± 0.34	0.48 ^hilnop^ ± 0.07	43.37 ^a^ ± 0.21	33.54 ^b^ ± 0.33	47.61 ^a^ ± 0.22
	PN 105	ND	ND	0.25 ^q^ ± 0.08	19.63 ^j^ ± 0.31	17.69 ^lm^ ± 0.17
	PN 145	29.32 ^f^ ± 0.23	0.44 ^mn^ ± 0.05	30.90 ^b^ ± 0.15	6.61 ^no^ ± 0.36	13.66 ^n^ ±0.18
*L. rhamnosus*	PN 14	0.44 ^l^ ± 0.07	0.83 ^efo^ ± 0.09	7.75 ^n^ ± 0.17	23.20 ^g^ ± 0.18	17.78 ^lm^ ± 0.19
	PN 108	0.76 ^l^ ± 0.07	0.39 ^mp^ ± 0.07	1.50 ^p^ ± 0.30	6.58 ^noq^ ± 0.21	6.48 ^r^ ± 0.22

ND: not detected. Results are reported as mean and standard deviation of three replicates. Aminopeptidase activity was expressed as the number of activity units per milligram of protein per minute. One unit of aminopeptidase activity was considered as the enzyme amount able to determine an increase in absorbance of 0.001 units. Different letters (a–p) in the same column indicate significant differences by one-way ANOVA test, followed by Tukey’s post-hoc test (*p* < 0.05).

**Table 4:** Microbial counts expressed as log_10_ cfu/mL and standard deviation (SD) of the main microbial groups detected in experimental (ECh) and control (CCh) cheeses at 0, 30 and 60 days of ripening.

Media	ECh			CCh		
	0	30	60	0	30	60
PCA	9.50 ^d^ ± 0.14	8.93 ^c^ ± 0.07	8.63 ^bc^ ± 0.08	8.34 ^b^ ± 0.05	8.84 ^c^ ± 0.07	7.96 ^a^ ± 0.10
MRS	9.29 ^c^ ± 0.07	8.77 ^b^ ± 0.14	8.74 ^b^ ± 0.06	7.80 ^a^ ± 0.05	7.88 ^a^ ± 0.10	7.63 ^a^ ± 0.09
VRBA *	1.19 ^ab^ ± 0.11	1.36 ^b^ ± 0.09	1.04 ^a^ ± 0.07	1.73 ^c^ ± 0.07	1.85 ^c^ ± 0.09	1.09 ^ab^ ± 0.08
M17	9.23 ^d^ ± 0.06	8.87 ^c^ ± 0.09	8.13 ^b^ ± 0.06	7.72 ^a^ ± 0.07	7.84 ^a^ ± 0.07	7.60 ^a^ ± 0.10
SDA	2.86 ^b^ ± 0.07	2.30 ^a^ ± 0.13	2.27 ^a^ ± 0.07	2.32 ^a^ ± 0.08	2.26 ^a^ ± 0.07	2.18 ^a^ ± 0.07
MSA	1.64 ^b^ ± 0.10	1.13 ^a^ ± 0.09	1.04 ^a^ ± 0.07	2.29 ^c^ ± 0.12	1.24 ^a^ ± 0.06	1.03 ^a^ ± 0.09
KAA	5.23 ^a^ ± 0.09	5.38 ^a^ ± 0.11	5.31 ^a^ ± 0.06	5.26 ^a^ ± 0.10	5.34 ^a^ ± 0.09	5.28 ^a^ ± 0.06

* incubated at 37 °C. Plate Count Agar (PCA), de Man Rogosa and Sharpe (MRS), Violet Red Bile Agar (VRBA), M17 Agar (M17), Sabouraud dextrose agar (SDA), Mannitol salt agar (MSA), Kanamycin Aesculin Azide agar (KAA). Different lowercase letters (a–d) in the same row, indicate a significant difference among experimental (ECh) or control (CCh) cheese samples at *p* < 0.05 (ANOVA with Tukey’s post-hoc test).

**Table 5:** Physico-chemical characteristics of experimental (ECh) and control (CCh) cheeses at 0, 30 and 60 days of ripening.

Parameters	ECh			CCh		
	0	30	60	0	30	60
pH	5.65 ^b^ ± 0.02	5.52 ^b^ ± 0.01	5.43 ^a^ ± 0.08	5.81 ^b^ ± 0.03	5.66 ^a^ ± 0.01	5.48 ^a^ ± 0.10
Moisture	44.81 ^c^ ± 0.15	40.76 ^b^ ± 0.03	34.58 ^a^ ± 0.05	44.25 ^c^ ± 0.12	40.88 ^b^ ± 1.09	34.86 ^a^ ± 1.29
Protein	26.98 ^a^ ± 0.22	28.66 ^a^ ± 0.08	30.08 ^b^ ± 0.06	27.25 ^a^ ± 0.14	28.34 ^a^ ± 0.44	29.80 ^a^ ± 0.59
Fat	23.56 ^a^ ± 0.11	25.78 ^a^ ± 0.03	30.03 ^b^ ± 0.04	23.62 ^a^ ± 0.19	25.90 ^a^ ± 0.34	29.90 ^b^ ± 0.70
Salt	1.54 ^a^ ± 0.20	2.01 ^b^ ± 0.02	1.83 ^b^ ± 0.01	1.40 ^a^ ± 0.04	2.04 ^b^ ± 0.03	1.91 ^b^ ± 0.02
Ash	2.89 ^a^ ± 0.28	2.88 ^a^ ± 0.03	3.33 ^b^ ± 0.01	2.78 ^a^ ± 0.03	2.70 ^a^ ± 0.07	3.47 ^b^ ± 0.08

Different lowercase letters (a–c) in the same row, indicate a significant difference among experimental (ECh) or control (CCh) cheese samples at *p* < 0.05 (ANOVA with Tukey’s post-hoc test).

**Table 6:** Volatile organic compounds (VOCs) (mg/kg) identified in experimental (ECh) and control (CCh) cheeses at 0, 30 and 60 days of ripening. Data are the means and standard deviations of three independent experiments analysed in duplicate.

	Cheese Samples					
VOCs	ECh			CCh		
	0	30	60	0	30	60
**Organic acids**						
Hexanoic acid	5.67 ^d^ ± 0.007	17.23 ^c^ ± 2.616	49.67 ^a^ ± 3.714	9.61 ^d^ ± 0.662	15.37 ^c^ ± 0.556	36.65 ^b^ ± 1.647
Butanoic acid	2.14 ^d^ ± 0.007	11.70 ^c^ ± 1.680	38.21 ^a^ ± 2.454	4.53 ^d^ ± 0.363	9.07 ^c^ ± 0.597	20.17 ^b^ ± 0.950
Octanoic acid	2.04 ^c^ ± 0.144	6.02 ^b^ ± 0.960	18.62 ^a^ ± 3.102	3.68 ^bc^ ± 0.271	5.44 ^bc^ ± 0.135	20.62 ^a^ ± 0.596
Acetic acid	0.59 ^d^ ± 0.095	5.00 ^bc^ ± 0.904	18.49 ^a^ ± 2.221	1.59 ^d^ ± 0.165	2.75 ^cd^ ± 0.117	7.27 ^b^ ± 0.800
n-Decanoic acid	0.89 ^b^ ± 0.063	2.57 ^b^ ± 0.421	8.10 ^a^ ± 1.809	1.21 ^b^ ± 0.075	1.91 ^b^ ± 0.169	7.63 ^a^ ± 0.341
Propanoic acid	ND	0.74 ^b^ ± 0.387	1.91 ^a^ ± 0.868	ND	0.03 ^b^ ± 0.005	0.39 ^b^ ± 0.010
Nonanoic acid	0.10 ^c^ ± 0.052	0.38 ^bc^ ± 0.133	0.79 ^a^ ± 0.008	0.25 ^c^ ± 0.015	0.71 ^ab^ ± 0.287	0.83 ^a^ ± 0.032
Heptanoic acid	0.07 ^c^ ± 0.002	0.23 ^b^ ± 0.043	0.72 ^a^ ± 0.109	0.16 ^c^ ± 0.02 b	0.24 ^b^ ± 0.006	0.81 ^a^ ± 0.053
3-Methylbutanoic acid	0.01 ^a^ ± 0.007	0.57 ^a^ ± 0.516	1.44 ^a^ ± 1.342	ND	0.04 ^a^ ± 0.006	ND
Pentanoic acid	0.06 ^d^ ± 0.002	0.26 ^c^ ± 0.041	0.76 ^a^ ± 0.049	0.09 ^d^ ± 0.003	0.21 ^c^ ± 0.020	0.47 ^b^ ± 0.016
Dodecanoic acid	0.07 ^b^ ± 0.007	0.19 ^b^ ± 0.027	0.65 ^a^ ± 0.162	0.10 ^b^ ± 0.006	0.12 ^b^ ± 0.004	0.69 ^a^ ± 0.058
9-Decenoic acid	0.07 ^b^ ± 0.011	0.21 ^b^ ± 0.035	0.69 ^a^ ± 0.179	0.09 ^b^ ± 0.012	0.13 ^b^ ± 0.009	0.62 ^a^ ± 0.012
**Alcohols**						
Ethanol	0.30 ^b^ ± 0.068	1.30 ^b^ ± 0.130	4.58 ^a^ ± 0.812	0.71 ^b^ ± 0.054	0.37 ^b^ ± 0.016	7.80 ^a^ ± 0.970
3-Methyl-1-butanol	0.15 ^d^ ± 0.026	1.12 ^b^ ± 0.315	2.76 ^a^ ± 0.800	0.24 ^cd^ ± 0.004	0.23 ^cd^ ± 0.026	0.15 ^d^ ± 0.046
Phenylethyl alcohol	0.20 ^b^ ± 0.018	1.21 ^ab^ ± 0.947	2.36 ^a^ ± 1.470	0.19 ^b^ ± 0.006	0.24 ^b^ ± 0.001	0.35 ^b^ ± 0.007
Benzyl alcohol	0.12 ^b^ ± 0.018	0.32 ^b^ ± 0.044	1.14 ^a^ ± 0.423	0.17 ^b^ ± 0.048	0.37 ^b^ ± 0.111	0.44 ^b^ ± 0.090
2-Butanol	ND	0.07 ^c^ ± 0.003	0.15 ^b^ ± 0.009	ND	0.18 ^a^ ± 0.013	0.06 ^c^ ± 0.002
1-Butanol	ND	0.07 ^c^ ± 0.006	0.17 ^a^ ± 0.010	ND	0.02 ^d^ ± 0.002	0.11 ^b^ ± 0.007
2,3-Butanediol	ND	0.04 ^c^ ± 0.008	0.19 ^a^ ± 0.030	ND	ND	0.07 ^b^ ± 0.007
1-Hexanol	0.01 ^e^ ± 0.006	0.03 ^c^ ± 0.000	0.08 ^b^ ± 0.009	0.02 ^de^ ± 0.001	0.02 ^cd^ ± 0.002	0.12 ^a^ ± 0.002
Phenol	0.01 ^a^ ± 0.007	0.01 ^a^ ± 0.013	ND	0.01 ^a^ ± 0.006	0.01 ^a^ ± 0.010	ND
**Esters**						
Ethyl hexanoate	0.11 ^d^ ± 0.012	10.12 ^c^ ± 1.033	34.23 ^a^ ± 4.790	0.47 ^d^ ± 0.039	1.47 ^d^ ± 0.139	19.8 ^b^ ± 0.682
Ethyl octanoate	0.08 ^d^ ± 0.002	3.98 ^c^ ± 0.023	14.14 ^a^ ± 0.523	0.14 ^d^ ± 0.014	0.52 ^d^ ± 0.002	9.31 ^b^ ± 0.808
Ethyl butanoate	0.03 ^d^ ± 0.002	0.65 ^c^ ± 0.049	5.16 ^a^ ± 0.044	0.06 ^d^ ± 0.002	0.14 ^d^ ± 0.018	2.12 ^b^ ± 0.286
Ethyl decanoate	0.04 ^d^ ± 0.003	0.57 ^c^ ± 0.030	2.74 ^a^ ± 0.081	0.04 ^d^ ± 0.001	0.07 ^d^ ± 0.003	1.65 ^b^ ± 0.171
Propyl hexanoate	ND	0.68 ^b^ ± 0.006	2.08 ^a^ ± 0.180	ND	0.02 ^c^ ± 0.005	0.69 ^b^ ± 0.042
Ethyl acetate	0.07 ^c^ ± 0.021	0.50 ^b^ ± 0.008	1.14 ^a^ ± 0.197	0.05 ^c^ ± 0.005	0.05 ^c^ ± 0.007	0.16 ^c^ ± 0.010
Ethyl n-dodecanoate	ND	0.26 ^c^ ± 0.015	0.90 ^a^ ± 0.100	ND	ND	0.57 ^b^ ± 0.044
Ethyl propanoate	ND	0.33 ^b^ ± 0.016	1.06 ^a^ ± 0.135	ND	ND	ND
3-Methylbutyl acetate	ND	0.40 ^b^ ± 0.177	0.76 ^a^ ± 0.131	ND	ND	0.22 ^b^ ± 0.038
Ethyl hexanoate	ND	0.15 ^c^ ± 0.003	0.58 ^a^ ± 0.023	ND	ND	0.39 ^b^ ± 0.045
2-Butanol acetate	ND	0.26 ^b^ ± 0.026	0.56 ^a^ ± 0.010	ND	ND	ND
Propyl octanoate	ND	0.12 ^c^ ± 0.006	0.46 ^a^ ± 0.011	ND	ND	0.21 ^b^ ± 0.004
Methyl isobutyrate	ND	0.03 ^c^ ± 0.000	0.37 ^a^ ± 0.029	ND	ND	0.23 ^b^ ± 0.022
Ethyl 9-decenoate	ND	0.07 ^c^ ± 0.008	0.34 ^a^ ± 0.016	ND	ND	0.18 ^b^ ± 0.018
Phenylethyl acetate	0.01 ^a^ ± 0.007	0.15 ^a^ ± 0.102	0.14 ^a^ ± 0.105	ND	ND	ND
Ethyl nonanoate	ND	0.0 ^c^ ± 0.005	0.14 ^a^ ± 0.001	ND	ND	0.07 ^b^ ± 0.011
Ethyl pentanoate	ND	0.01 ^b^ ± 0.012	0.10 ^a^ ± 0.007	ND	ND	0.08 ^c^ ± 0.007
Propyl decanoate	ND	0.02 ^c^ ± 0.001	0.08 ^a^ ± 0.013	ND	ND	0.05 ^b^ ± 0.000
3-Methylbutyl butanoate	ND	0.02 ^a^ ± 0.015	0.07 ^a^ ± 0.072	ND	ND	ND
**Ketones**						
2-Butanone	ND	0.10 ^b^ ± 0.016	0.30 ^a^ ± 0.059	ND	0.21 ^c^ ± 0.039	ND
Diacetyl	ND	0.59 ^b^ ± 0.03	1.42 ^a^ ± 0.280	ND	0.16 ^c^ ± 0.160	ND
Acetoin	0.10 ^c^ ± 0.018	0.24 ^b^ ± 0.048	0.59 ^a^ ± 0.064	0.04 ^c^ ± 0.008	0.48 ^a^ ± 0.073	ND
2-Nonanone	0.04 ^b^ ± 0.002	0.07 ^b^ ± 0.018	0.41 ^a^ ± 0.291	0.11 ^ab^ ± 0.028	0.41 ^a^ ± 0.001	0.03 ^b^ ± 0.030
2-Heptanone	0.04 ^b^ ± 0.009	0.04 ^b^ ± 0.002	0.39 ^a^ ± 0.194	ND	ND	ND
2-Undecanone	ND	0.01 ^c^ ± 0.009	0.04 ^a^ ± 0.003	0.01 ^c^ ± 0.005	0.03 ^b^ ± 0.002	ND
**Aldehydes**						
Hexanal	0.05 ^a^ ± 0.012	ND	ND	0.03 ^b^ ± 0.001	ND	ND
Benzaldehyde	ND	0.03 ^b^ ± 0.014	0.07 ^a^ ± 0.001	ND	ND	ND
**Terpenes**						
beta-Pinene	ND	ND	ND	0.11 ^a^ ± 0.008	0.05 ^b^ ± 0.004	ND
d-Limonene	0.23 ^cd^ ± 0.092	0.27 ^c^ ± 0.109	0.16 ^cd^ ± 0.005	1.53 ^a^ ± 0.034	1.15 ^b^ ± 0.128	0.04 ^d^ ± 0.039
**Others**						
6-Pentyl-2H-pyran-2-one	0.03 ^cd^ ± 0.003	0.05 ^c^ ± 0.012	0.15 ^a^ ± 0.02	0.04 ^d^ ± 0.001	0.05 ^c^ ± 0.007	0.10 ^b^ ± 0.001

a–e: values in the same line with different superscript letters differ significantly (*p* < 0.05) based on one-way ANOVA followed by Tukey’s post-hoc tests. ND not detected.

## Data Availability

Not applicable.

## Abbreviations

CCh	control cheese
CFE	cell free extract
ECh	experimental cheese
EPS	exopolysaccharides
FFAs	free fatty acids
LAB	lactic acid bacteria
MALDI-TOF MS	matrix-assisted laser desorption/ionisation time-of-flight mass spectrometry
MIC	minimum inhibitory concentration
NSLAB	non-starter LAB
PDO	Protected Designation of Origin
PN	Provola dei Nebrodi
pNA	p-nitroanilide
QPS	qualified presumption of safety
SLAB	starter LAB
VOCs	volatile organic compounds
